# Designing electrolytes by thermodynamics

**DOI:** 10.1093/nsr/nwaf100

**Published:** 2025-03-17

**Authors:** Yaozu Wang, Huicong Yang, Tianzhao Hu, Nan Piao, Feng Li, Hui-Ming Cheng

**Affiliations:** School of Materials Science and Engineering, University of Science and Technology of China, Shenyang 110016, China; Shenyang National Laboratory for Materials Science, Institute of Metal Research, Chinese Academy of Sciences, Shenyang 110016, China; School of Materials Science and Engineering, University of Science and Technology of China, Shenyang 110016, China; Shenyang National Laboratory for Materials Science, Institute of Metal Research, Chinese Academy of Sciences, Shenyang 110016, China; School of Materials Science and Engineering, University of Science and Technology of China, Shenyang 110016, China; Shenyang National Laboratory for Materials Science, Institute of Metal Research, Chinese Academy of Sciences, Shenyang 110016, China; School of Materials Science and Engineering, University of Science and Technology of China, Shenyang 110016, China; Shenyang National Laboratory for Materials Science, Institute of Metal Research, Chinese Academy of Sciences, Shenyang 110016, China; School of Materials Science and Engineering, University of Science and Technology of China, Shenyang 110016, China; Shenyang National Laboratory for Materials Science, Institute of Metal Research, Chinese Academy of Sciences, Shenyang 110016, China; Shenzhen Key Lab of Energy Materials for Carbon Neutrality, Institute of Technology for Carbon Neutrality, Shenzhen Institute of Advanced Technology, Chinese Academy of Sciences, Shenzhen 518055, China; Faculty of Materials Science and Energy Engineering, Shenzhen University of Advanced Technology, Shenzhen 518000, China

**Keywords:** electrolytes, solvation structure, thermodynamic theory, competitive equilibrium, enthalpy, entropy

## Abstract

The immature design theory of electrolytes limits their targeted solvation structure formation and application in batteries. Here, based on the precondition that an electrolyte or solution is a system at a thermodynamic equilibrium state, we try to develop a thermodynamic theory to guide the electrolyte solvation structure design. In this theory, thermodynamic competitive equilibrium between cation-solvent interaction and cation-anion interaction, and between enthalpy and entropy, are two key points determining solute dissolution and formation of various solvation structures. Using this thermodynamic competitive equilibrium theory, the essential principle of all the recently developed electrolyte systems such as high concentration electrolyte, localized high concentration electrolyte, weak solvated electrolyte, anion coordination electrolyte and high-entropy electrolytes can be perfectly explained. We hope that this theory can help accelerate the development of electrolyte study, and enlighten the emergence of advanced electrolytes with unique solvation structures and attractive properties.

## Teaser

Based on the recognition that electrolytes are thermodynamic equilibrium systems, this study establishes a thermodynamic competitive equilibrium theory according to the energy criteria, providing theoretical guidance for designing advanced electrolytes.

## INTRODUCTION

**Figure 1.**
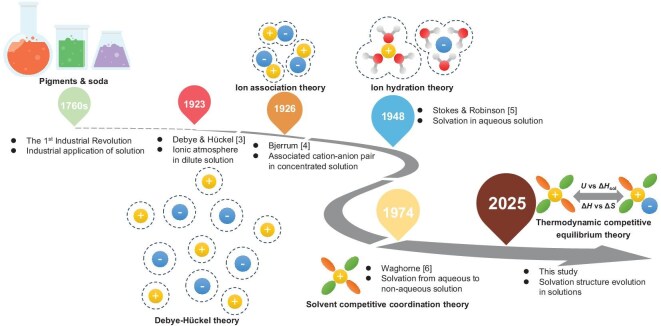
History of the development of the solvation theory.

Solution is ubiquitous and plays an important role in life and in the fields of chemistry, metallurgy, environmental protection, energy and others. The solvation structure in a solution determines its physical and chemical properties, such as conductivity, viscosity, etc., as well as the thermodynamic and kinetic characteristics of chemical reactions occurring in the solution including the direction, rate and limit of the reactions. However, looking back at the history of solvation structure study of aqueous and non-aqueous solutions, the development of solution theory is far behind the application of reactions in solutions (Fig. 1). Since the Industrial Revolution in the 1760s, solution reactions have been used to manufacture industrial pigments, soda and other products, which has brought great changes to human production and life [1,2]. However, it was not until 1923 that the Debye–Hückel theory first proposed the discussion of the microstructure of electrolyte solutions, but it only involved ion-ion long-range electrostatic attraction and ignored ion-ion short-range repulsion [3]. Subsequently, in 1926, Bjerrum proposed the ion association theory based on a concept that ion pairs form and move together because of the Coulomb force between positive and negative ions [4]. In 1948, Stokes and Robinson considered the interaction between ions and water molecules and proposed the ion hydration theory, in which ions do not exist independently, but form solvated ions through ion-solvent interactions [5]. Then moving forward to 1974, Waghorne proposed the latest model of solvent competitive coordination of ionic solvation based on the formation energy of ionic and solvent complexes, which can be extended from water to non-aqueous electrolytes, and from unary to binary solvent solutions [6]. This study of solvation structures comes back to its thermodynamics, that is, energy is the essence guiding the solvation structure design of the solution. However, the theoretical knowledge about the thermodynamics of solvation structures at present remains in books, and how to apply it in the development of electrolyte solutions in daily life and industry is still a challenge.

This problem is prevalent in energy storage and conversion research fields such as batteries, capacitors, electrolytic cells, etc., resulting in the current electrolyte design mainly following a ‘trial and error’ method or empirical attempt [7,8]. The device development cycle is long and the cost is high. Taking the most widely used lithium-ion batteries as an example, the electrolyte used is a typical solution composed of cations and anions from lithium salts (LiPF_6_, etc.), solvents (ester and ether solvents, etc.) and additives [9]. To meet the application needs of various scenarios, numerous electrolyte systems are constantly being developed, from routine concentration electrolytes to high-concentration electrolytes, localized high-concentration electrolytes, weak solvated electrolytes and high-entropy electrolytes [10–13]. These electrolyte systems display widely varying solvation structures, reflecting the different nature and strength of the interactions among cations, anions and solvent molecules, such as ion-ion interactions, ion-dipole interactions, dipole-dipole interactions, hydrogen bond and van der Waals forces, determining the final performance of batteries under different application scenarios [14,15]. However, electrolyte systems still lack a unified theory, which can help us more deeply understand the formation and transformation of existing solvation structures, and guide us to a better design for new solvation structures in order to obtain various desired performances.

We believe that as a solution, random Brownian motion leads an electrolyte containing solvents, cations and anions to a thermodynamic equilibrium system. Therefore, solvation structure thermodynamics based on Gibbs free energy (∆*G*) provides an opportunity to quantitatively assess the solvation structure characteristics of all electrolyte systems, regardless of the electrolyte composition. According to the second law of thermodynamics, ∆*G* is determined by the enthalpy (∆*H*), temperature (*T*) and entropy (∆*S*) of the system: ∆*G =* ∆*H − T*∆*S* [16,17].

In this theoretical study, we explore the thermodynamics and energies in the formation of solvation structures, point out different intrinsic driving forces that change the solvation structure from a thermodynamic aspect, and finally elucidate thermodynamic principles in order to achieve the desired solvation structure. We believe this study can provide a profound, concrete, and varied understanding of the solvation structures of electrolytes from a fundamental thermodynamic point of view, thereby bringing the electrolyte and solution design theory closer to real application scenarios not only for batteries, but other solution reactions as well.

## RESULTS AND DISCUSSION

### Dissolution thermodynamics of a solute

**Figure 2.**
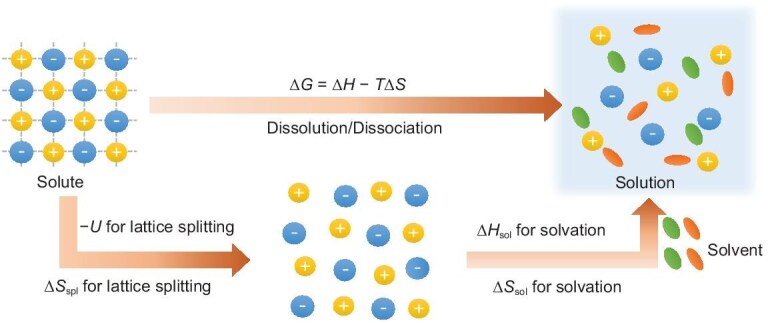
Thermodynamics of solution formation.

The first prerequisite for electrolytes or solutions is the dissolution of a solute (such as lithium salts) in a solvent to form a homogeneous phase. This process generally involves lattice dissociation (splitting) of the salt and solvation of the cation. Specifically, solvent molecules accumulate around ions through ion-solvent interactions, releasing solvation energy (∆*H*_sol_) to overcome the lattice energy (*U*), that is the cation-anion electrostatic interaction of the solute (Fig. 2). The change of Gibbs free energy (∆*G*) during dissolution is determined by the enthalpy change (∆*H*) and the entropy change (∆*S*) of the system, and can be described by the Born–Haber cycle:


(1) \begin{eqnarray*}
\Delta G = {G_2} - {G_1} = \Delta H - T\Delta S.
\end{eqnarray*}

If we assume *G*_1_ is the initial state that contains separated solvent and solute, while *G*_2_ is the final state that represents a homogeneous solution, Δ*G* < 0 is necessary for the salt to dissolve spontaneously. This process obviously requires a more negative Δ*H* and a larger Δ*S* as shown in Equation (1). And according to Equation (2), Δ*H* originates from the influence of two parameters, *U* and Δ*H*_sol_:


(2) \begin{eqnarray*}
\Delta H = - U + \Delta {H_{{\mathrm{sol}}}}.
\end{eqnarray*}

To weaken *U* (or cation-anion electrostatic interactions), large anion groups are widely used in lithium batteries, such as PF_6_^−^, FSI^−^, TFSI^−^, DFOB^−^ and ClO_4_^−^, etc [18–21]. The interaction of such anions with other cations like Li^+^, Na^+^, etc., is significantly reduced due to the delocalization of negative charges (*z*) on the anions, which reduces *U* to increase the solubility of lithium salt according to Equation (3):


(3) \begin{eqnarray*}
{U_{{\mathrm{ion}} - {\mathrm{ion}}}} = - \frac{{{z_1}{z_2}{e^2}}}{{4\pi {\varepsilon _0}{\varepsilon _{{\mathrm{sol}}}}{r_1}}}.
\end{eqnarray*}

In contrast, lithium salts, such as LiF and LiCl, which have strong cation-anion electrostatic interaction, are nearly insoluble in aprotic solvents because of the larger *U* than LiPF_6_, LiFSI and LiTFSI, etc [22]. Besides *U*, Δ*H* is also affected by the solvation energy (Δ*H*_sol_) of the solvent, as it describes the strength of the interaction between the solvent and the cation as in Equation (4) [23]:


(4) \begin{eqnarray*}
{U_{{\mathrm{ion}}-{\mathrm{dipole}}}} = - \frac{{ze\mu {\mathrm{cos}}\theta }}{{4\pi {\varepsilon _0}{\varepsilon _{{\mathrm{sol}}}}r_2^2}}.
\end{eqnarray*}

In Equations (3) and (4), *ε*_0_ is the vacuum permittivity, *ε*_sol_ is the permittivity of the solvent, *z*_1_, *z*_2_ and *z* are ion charge, *r*_1_ is the distance between the positive charge center and negative charge center, *r*_2_ is the distance between the ion and the dipole center, *μ* is the dipole moment, and *θ* is the dipole angle. Equation (4) describes the physical parameters affecting the solvation energy. To more directly evaluate the solvation energy of one particular solvent, donor number (DN) is applied as shown in Equation (5) [24]:


(5) \begin{eqnarray*}
{\mathrm{D{:}}} &+& {\mathrm{SbC}}{{\mathrm{l}}_5} \leftrightarrow {\mathrm{D}} - {\mathrm{SbC}}{{\mathrm{l}}_5}; \\
&&{\mathrm{DN}}\,= - \Delta H\,\,\left( {{\mathrm{kcal\,\,mo}}{{\mathrm{l}}^{ - 1}}} \right).
\end{eqnarray*}

where D is the solvent with lone electron pairs, and the DN value is defined as the negative enthalpy value of the 1:1 coordination structure formed by the Lewis base solvent and the standard Lewis acid antimony pentachloride (SbCl_5_). Solvents with higher DN mean a more negative Δ*H*_sol_, causing Δ*H* in Equation (1) to become more negative and thus Δ*G* becomes more negative to promote solute dissolution. For example, one general strategy to promote the solubility of LiNO_3_ in lithium-metal battery electrolytes to increase the stability of the anode-electrolyte interface is selecting a new solvent with high DN to increase the interaction strength between Li^+^ and solvent, leading Δ*H*_sol_, Δ*H* and Δ*G* towards a more negative value [25–27].

When reconsidering Equations (3) and (4), it can be found that another key parameter that affects *U* and Δ*H*_sol_ is ignored, that is the permittivity of solvent, *ε*_sol_. Since *ε*_sol_ exists as a denominator of both Equations (3) and (4), it means that this physical property of solvents can increase or decrease both *U* and Δ*H*_sol_ equally and simultaneously. Therefore, *ε*_sol_ cannot change the positive or negative characteristic of Δ*H*, but can change the absolute value of Δ*H*. Once taking the other key parameter, entropy (∆*S*), into consideration, Δ*G* and the dissolution process of solutes can be changed since the dissolution of solutes is always an entropy-increasing process because of lattice splitting entropy (∆*S*_spl_) and solvation entropy (∆*S*_sol_) as shown in Equation (6):


(6) \begin{eqnarray*}
\Delta S = \Delta {S_{{\mathrm{spl}}}} + \Delta {S_{{\mathrm{sol}}}}.
\end{eqnarray*}

Once the solvent has quite a high permittivity, the absolute value of Δ*H* can reach a quite small degree. And no matter whether Δ*H* is positive or negative, the tendency of ‘entropy increase’ can lead to a negative ∆*G* and the dissolution of solutes. For example, one strategy to promote the dissolution of LiNO_3_ is selecting solvents with a high permittivity [28,29].

In summary, a solvent with a higher DN means that Δ*H* is more negative and Δ*G* is more negative, which is more intuitive but lacks the consideration of Δ*S*. The high permittivity of the solvent decreases both the cation-anion interaction and the cation-solvent interaction, and the Δ*H* becomes more positive, which is the main difference with DN. But the cations and anions move more freely, which increases the Δ*S* of the solution system and makes the Δ*G* more negative. Solvents with both high DN value and high permittivity, such as dimethyl sulfoxide (DMSO), *N,N*-dimethylformamide (DMF), tetramethylurea (TMU), etc., can dissolve more LiNO_3_ (>4 mol L^−1^), as shown in Table S1. However, both DN and permittivity have certain limitations, and a more accurate descriptor is needed to establish a quantitative relationship between salt solubility and the thermodynamic properties of the solvents.

### Thermodynamic competitive equilibrium of a solvation structure

**Figure 3.**
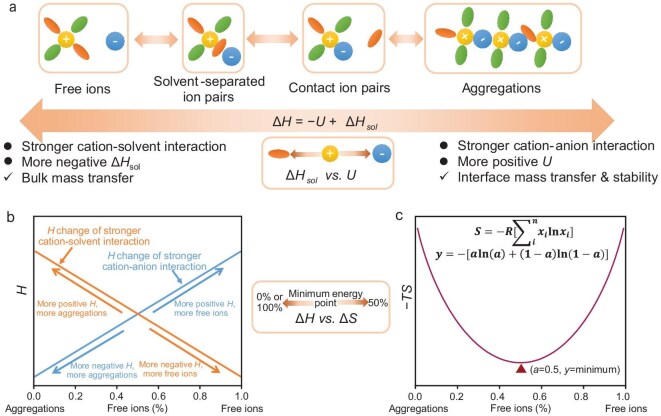
(a) Illustration of different singular solvation structures and their competitive equilibrium by enthalpy. (b) Illustration of enthalpy change from solvation structure conversion under different relative cation-solvent and cation-anion interaction strengths. The orange curve indicates that the cation-solvent interaction is stronger than the cation-anion interaction. The blue curve indicates that the cation-solvent interaction is weaker than the cation-anion interaction. (c) Illustration of entropy change from solvation structure conversion.

Once a solute is dissolved in a solvent, the cation is surrounded by solvent molecules and anions, and forms complexes known as a ‘solvation structure’. Based on the different amounts of solvent molecules and anions around cations, the solvation structures can be separated into four categories: free ions, solvent-separated ion pairs, contact ion pairs and aggregations (Fig. 3a) [30]. Since the solvated cations are the species directly taking part in electrochemical reactions at the electrode-electrolyte interface, obtaining an aimed solvation structure is necessary for electrolyte design. For example, a solvation structure with more free ions is beneficial for bulk ion transfer due to the increased dissociation degree of solute, while a solvation structure with more contact ion pairs or aggregations is beneficial for interface ion transfer and stability by the fast desolvation process and redox products of anions [31,32]. Establishing a thermodynamical principle to guide the solvation structure formation in electrolytes is quite reasonable since an electrolyte is always a solution under a thermodynamic equilibrium state. Like the dissolution process, Equation (1) is also suitable for guiding the solvation structure so that the formation of different solvation structures always follows the same principle that realizes a minimum Gibbs free energy of the solution.

When comparing different solvation structures, it can be found that in the limited coordination space around cations, solvents and anions are in a competitive relationship and this competitive relationship can be described by Equation (2). Therefore, the enthalpy factor is valuable and often considered when modifying solvation structures in an electrolyte [33]. For example, once taking a single solvation structure containing only one cation and several solvents/anions into consideration, and assuming that the initial state contains these species without any interactions between each other, then considering the cation-solvent interaction and cation-anion interaction inside the initial state, a stronger cation-solvent interaction can lead to a higher tendency to form a solvation structure with free ions as the final state, while a stronger cation-anion interaction leads to a higher tendency to form a solvation structure with contact ion pairs or aggregations as the final state (Fig. 3b). These two final states have different solvation structures, but both of them have the minimum Gibbs free energy under their own interaction strength situation.

However, when this example model is enlarged from one single solvation structure to a real electrolyte containing a large amount of cations, anions and solvents, the final state is quite complex and different, because system entropy should be considered. The enthalpy change has told us that it tends to form only one kind of solvation structure towards the minimum Gibbs free energy. But only one kind of solvation structure obviously decreases the system entropy according to the physical definition of entropy [34,35]:


(7) \begin{eqnarray*}
S = - R\left[ {\mathop \sum \limits_i^n {x_i}{\mathrm{ln}}{x_i}} \right].
\end{eqnarray*}

Here, ${x_i}$ refers to the proportion of *i* state in the electrolyte. The decrease or vanishing of various states, or in other words, the decrease of system disorder degree, leads to an increase in Gibbs free energy. For example, considering a system containing only two kinds of states, State a (free ions solvation structure) and State b (aggregations solvation structure), the mathematic expression of the entropy is calculated as follows:


(8) \begin{eqnarray*}
a + b = 1,
\end{eqnarray*}

(9) \begin{eqnarray*}
S &=& - R\left[ {a{\mathrm{ln}}a + b{\mathrm{ln}}b} \right] \\
&=& - R\left[ {a{\mathrm{ln}}a + \left( {1 - a} \right)\ln \left( {1 - a} \right)} \right].
\end{eqnarray*}

According to the functional image of Equation (8), only *a* = *b* = 0.5 can realize the maximum entropy (or minimum *− TS*, Fig. 3c), as well as the minimum Gibbs free energy according to Equation (1). Here comes a paradox that enthalpy aims to realize the minimum Gibbs free energy by forming a single solvation structure, while entropy aims to realize the minimum Gibbs free energy by forming the same amount of various solvation structures. In other words, enthalpy describes the competition among different solvation structures through the competitive relationship between cation-solvent interaction and cation-anion interaction, while entropy prevents this competition of reaching equilibrium of various solvation structures. Therefore, in the final state of a using an electrolyte, various solvation structures coexist, with only one or two kinds dominant. When needing to change the dominant solvation structure species, modifying the relative strength (competitive relationship) of cation-solvent interaction and cation-anion interaction to shift the original thermodynamic equilibrium to a new one is a good choice. To help better understand the solvation structure change caused by these thermodynamic parameters, the thermodynamic parameters of common solvents and salts are shown in Tables S2 and S3. And these thermodynamic parameters can also be obtained by experimental methods and theoretical calculation methods [36,37].

### Designing electrolyte solvation structures by thermodynamics

Since the thermodynamic principle of electrolytes has been established in the above sections, designing an electrolyte with a specular solvation structure and properties might be much easier following this thermodynamic competitive equilibrium guideline. In recent decades, many new concepts of electrolytes have been proposed, such as high concentration electrolyte, localized high concentration electrolyte, weak solvated electrolyte, anion coordination electrolyte and high-entropy electrolyte [38–40]. Even though these new electrolytes and new designing methods are quite different from conventional electrolytes (1 mol L^−1^ LiPF_6_ in ethylene carbonate (EC) and dimethyl carbonate/diethyl carbonate/ethyl methyl carbonate (DMC/DEC/EMC), or 1 mol L^−1^ LiTFSI in 1,2-dimethoxyethane/1,3-dioxolane (DME/DOL)), they have the same goal of increasing anion coordination in solvation structures because this anion-rich and solvent-lean structure has two main advantages. First, this solvation structure can realize a fast desolvation process to accelerate charge transfer kinetics [41]. And second this solvation structure can realize an inorganic components-rich electrode-electrolyte interface layer by the redox of anions to increase interface stability and increase ion transport speed inside the electrode-electrolyte interface layer [42]. We found that all these electrolyte designs can be included in our thermodynamic competitive equilibrium theory.

It should be pointed out that, in essence, all these electrolytes follow the same design, that is, pursuing a critical solvation structure state. This critical state has a saturated solute in solvent since anion coordination has a threshold beyond which the solute precipitates. The Gibbs free energy of this critical state is defined as *G*_cri_. The Gibbs free energy of a common electrolyte, such as 1 mol L^−1^ LiPF_6_ in EC/DMC is defined as *G*_com_. Notably, due to the strong solvation ability of EC and DMC, the Li^+^-solvent interaction is much greater than the Li^+^-anion interaction, so the Li^+^-solvent coordination releases more energy, and the Δ*H*_sol_ is more negative thus resulting in the lowest Gibbs free energy. Therefore, the solvation structure is mainly dominated by free ions and solvent separated ion pairs, which means that anions are mainly excluded from the solvation sheath. Then we define a descriptor $\vartheta $ referring to the Gibbs free energy discrepancy between a common electrolyte and a new concept electrolyte:


(10) \begin{eqnarray*}
\vartheta = {\mathrm{\Delta }}{G_{{\mathrm{cri}}}} - {\mathrm{\Delta }}{G_{{\mathrm{com}}}}.
\end{eqnarray*}

**Figure 4.**
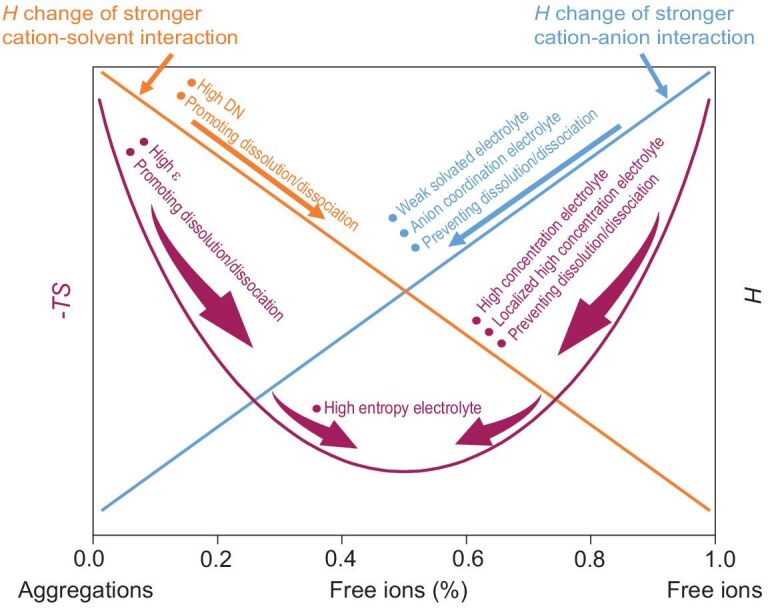
Different electrolyte design strategies by the thermodynamic competitive equilibrium theory.

The energy aim of new electrolytes is overcoming $\vartheta $ to form anion-rich solvation structures by both enthalpy strategies and entropy strategies. The basic thermodynamic principles are explained in the following sections and are summarized in Fig. 4 and Table S4. Besides, strategies for promoting solute dissolution/dissociation mentioned in this section can also be explained by the left side orange curve and purple curve in Fig. 4.

### Designing the solvation structures of electrolytes by enthalpy

According to the thermodynamic competitive equilibrium theory of solvation structures mentioned in the previous section, modifying enthalpy is a reasonable way to change the original competitive equilibrium, that is, modifying the dominant solvation structures in electrolytes.

According to Equation (2), once an anion-rich solvation structure is pursued, there are always two ways of doing so: making Δ*H*_sol_ less negative and making *U* more positive. A weak solvated electrolyte follows the less negative Δ*H*_sol_ strategy by selecting a low DN solvent according to Equation (5) or reducing the polarity through solvent molecular design according to Equation (4). The essence of these strategies is weaking the oxygen coordination bonds between solvents and cations by decreasing the electron cloud density around the coordinated oxygen atoms (Fig. 4, blue curve, the first point). Our previous work has given out a solvent molecular design paradigm by fluorination [43]. When a solvent is fluorinated, cation-solvent interaction becomes weaker and the relative strengths of cation-solvent and cation-anion interactions are altered. The formation of a cation-anion coordination structure causes more negative enthalpy release than the formation of the cation-solvent coordination structure, thereby forming a more anion-rich solvation structure to overcome the Gibbs free energy discrepancy $\vartheta $ and achieving the lowest Gibbs free energy. Enlarging the spatial structure of a coordinated solvent is another way to weaken the interaction between solvents and cations to shift the thermodynamic competitive equilibrium of solvation structures. For example, Cui's group has compared the solvation structure difference between the electrolytes using 1,2-dimethoxyethane (DME) or 1,2-diethoxyethane (DEE) as solvents [44]. Even though a DEE molecule has a higher electron cloud density around the coordinated oxygen atoms and a higher DN than that of DME, the experimentally measured oxygen coordination bond between solvent and cation is weaker. This phenomenon is attributed to the large steric hindrance of the ethoxy group in DEE.

Making *U* more positive is another way of shifting the thermodynamic competitive equilibrium towards an anion-rich dominant solvation structure in electrolytes. According to Equation (3), enlarging the charge density of anions is a reasonable method. The selecting of anions can be referred to the DN of anions, similar to the DN of solvents. Liu's group designed a new kind of anion with functional groups containing oxygen atoms [45,46]. Besides the electrostatic interaction between cations and anions, this new kind of anion can construct oxygen coordination bonds between cations and anions to make *U* more positive in order to overcome the Gibbs free energy discrepancy $\vartheta $ and realize an anion-rich solvation structure in electrolytes (Fig. 4, blue curve, the second point). This anion coordination electrolyte opens a new horizon for electrolyte design.

### Designing the solvation structures of electrolytes by entropy

Unlike the enthalpy modification strategy that the energies of cation-solvent and cation-anion interaction are directly described by Equations (3) and (4), and measured by Equation (5), the strategy for designing the solvation structures of an electrolyte by entropy is nonobjective, since the concept of entropy is more nonobjective than that of enthalpy. In fact, high concentration electrolyte and localized high concentration electrolyte are two kinds of electrolytes following the entropy strategy (Fig. 4, purple curve).

For the conventional electrolyte, the solute concentration is generally 1 M, and the salt/solvent ratio is low to a mole ratio of 1:7–1:10 [47]. Cations are completely solvated, while a large amount of remaining free solvents and anions are excluded from the first solvation sheath, forming a solvation structure dominated by free ions or solvent-separated ion pairs. In a high concentration electrolyte, the cation-solvent interaction is stronger than the cation-anion interaction, so the cation-solvent coordination has a more negative solvation enthalpy, like that in common concentration electrolytes. Therefore, when only considering a single solvation structure, free ions or a solvent-separated ion pair is preferred. But in fact, at a high concentration (>4 mol L^−1^), solvents are not enough to fill the whole coordination space of all the cations. There are two situations. Situation 1 is that solvents take part in the solvation structure of all the cations equally, and the unsaturated solvation sites of each cation are replenished by anions to form contact ion pairs or an aggregation dominated solvation structure. Situation 2 is that solvents reach a saturated coordination around partial cations to form a solvation structure with free ions or solvent-separated ion pairs, but other cations cannot be dissolved since not enough solvents and precipitated solute exists in the electrolyte. Whether Situation 1 or Situation 2 is preferred depends on the Gibbs free energy. Since all the solvents take part in the solvation structure of cations, it can be concluded that Δ*H*_sol-1_ of Situation 1 equals to Δ*H*_sol-2_ of Situation 2. Moreover, all the solvents replace the anions around cations, consequently, *U*_1_ of Situation 1 equals to *U*_2_ of Situation 2. According to Equation (2), Δ*H*_1_ equals Δ*H*_2_ as well. However, entropy is quite different. Because of the existence of Δ*S*_spl_ and Δ*S*_sol_ according to Equation (6), Situation 1 obviously has a higher Δ*S*_1_ than Situation 2 since there is undissolved solute in Situation 2. Therefore, entropy leads to a lower Gibbs free energy in Situation 1 than that in Situation 2 and leads to the formation of a solvation structure with contact ion pairs or aggregations. Although a high concentration electrolyte can effectively achieve stable SEI and greatly improve electrode-electrolyte interface stability owing to the anion-rich solvation structure, it suffers from poor wettability, poor ionic conductivity and high cost [48]. To overcome these disadvantages, localized high concentration electrolytes have been developed [49]. Generally speaking, adding solvents with weak polarity (called a diluter) to dilute a high concentration electrolyte to ∼1 mol L^−1^ can form a localized high concentration electrolyte. The localized high concentration electrolyte follows the same thermodynamic principle of solvation structure formation due to two reasons. First, the diluter can hardly take part in or affect the solvation structure formed by solvents with high polarity because of its weak polarity. Second, the mole ratio of solute to high polarity solvent is almost the same as that of a high concentration electrolyte. Our recent findings also showed an additional role for diluters, that is weaking oxygen coordination bonds to make Δ*H* positive [50]. The hydrogen atoms of 1,1,2,2-tetrafluoroethyl-2,2,3,3-tetrafluoropropyl ether will have diluent-solvent interactions with the oxygen atoms of tetrahydrofuran and 2-methyltetrahydrofuran, thereby weakening the electronegativity of the oxygen atoms of the solvent and further increasing the competitiveness of cation-anion coordination, therefore the enthalpy and entropy reach a new state of competitive equilibrium.

Another regulation strategy using solvation entropy is similar to high-entropy alloy materials [51]. By using multiple components to enhance Δ*S*, it becomes possible to mitigate the impact of the increased Δ*H* and thereby reduce the Gibbs free energy, thus improving the stability and reaction rate of electrolytes (Fig. 4, purple curve). Accordingly, high-entropy electrolytes are implemented by integrating diverse components to achieve higher disorder and complexity. The flexibility of high-entropy electrolytes offers great potential for optimizing ion-transport conductivity, electrode-electrolyte interface properties and cell stability. Cui *et al*. varied the solvation entropy by increasing the diversity of molecules while selecting solvents with similar structures to minimize differences in solvation enthalpy [52]. Compared to electrolytes containing two kinds of solvents, an electrolyte containing five kinds of solvents significantly doubles the ionic conductivity while maintaining an anion-rich solvation structure. The high entropy of the electrolyte containing five kinds of solvents drives the reduction of clusters and the improvement of ionic conductivity.

## CONCLUSION

Based on the precondition that an electrolyte or a solution is a system at thermodynamic equilibrium state, we developed a thermodynamic theory to guide the design of electrolyte solvation structures. In our theory, two kinds of thermodynamic competitive equilibriums are important. One is the competitive equilibrium between cation-solvent interaction and cation-anion interaction, which determines enthalpy in electrolytes at the thermodynamic equilibrium state. The other is the competitive equilibrium between enthalpy and entropy, which determines the minimum Gibbs free energy of electrolytes at the thermodynamic equilibrium state. According to these two kinds of thermodynamic competitive equilibriums, the dissolution of solutes to form solutions can be well described. Furthermore, we pointed out that these two kinds of thermodynamic competitive equilibriums have the same thermodynamic essence which can explain the recently developed electrolyte systems such as high concentration electrolytes, localized high concentration electrolytes, weak solvated electrolytes, anion coordination electrolytes and high-entropy electrolytes. The essence is making anion-rich solvation structures presenting the minimum Gibbs free energy by enthalpy and entropy.

However, we admit to some deficiencies of this thermodynamic competitive equilibrium theory. First, this thermodynamic theory can only describe the formation of a dominant solvation structure in bulk and equilibrium state electrolytes. However, in the real operation scenario of a battery, the electrolyte is not at an equilibrium state or not even at a steady state, which means that the solvents, cations and anions are redistributing with time, current density, voltage and temperature, which may diverge the solvation structures dynamically from theoretical ones. Non-equilibrium thermodynamics will help to provide directions for the design of solvation structures under real dynamic conditions. Besides, for an electrochemical reaction occurring in a battery, the solvation structure at the electrode-electrolyte interface is more important than that in a bulk electrolyte. And the solvation structures at these two positions are obviously different because of chemical adsorption and electrostatic field [53,54]. It is also quite difficult to establish a theory or model to describe the transient solvation structures at the interface, therefore, theoretical calculations and *in-situ* interface characterization may help [55]. Second, this thermodynamic theory only considers coordination and electrostatic interactions, without taking weak interactions such as van der Waals’ force and hydrogen bond into consideration. In fact, these weak interactions can contribute a part of enthalpy, entropy and Gibbs free energy in a thermodynamic equilibrium system, thus affecting the properties of electrolytes. For example, our recent study has proven that the hydrogen bond inside an electrolyte can dramatically affect the stability of the electrolyte at high temperatures [56]. Therefore, optimizing our theory by taking weak interactions into consideration is necessary to better describe the thermodynamics and physicochemical properties of electrolytes. Third, we believe that thermodynamic competitive equilibrium between cation-solvent interaction and cation-anion interaction has its own energy limitation since both coordination bond and ionic bond have the same energy limitation, neither weaker than van der Waals’ force nor stronger than covalent bond. Therefore, designing new solvent and solute molecules to overcome this energy limitation is of great significance to widen the functions of electrolytes. Finally, we also hope our picture of thermodynamic competitive equilibrium theory can help researchers develop new concepts, new structures and new mechanisms in electrolyte studies.

## Supplementary Material

nwaf100_Supplemental_File
