# Supplementary material for: Designing electrolytes by thermodynamics
Source: Natl Sci Rev. 2025 Mar 17;12(5):nwaf100. doi: 10.1093/nsr/nwaf100 (PMC12016802; doi:10.1093/nsr/nwaf100)
Supplement: nwaf100_Supplemental_File [file nwaf100_supplemental_file.pdf]

Supplementary Data

Designing electrolytes by thermodynamics

Yaozu Wang[1, 2], Huicong Yang[1, 2*], Tianzhao Hu[1, 2], Nan Piao[1, 2], Feng Li[1, 2*] and Hui-Ming Cheng[3 ,4*]

[1] School of Materials Science and Engineering, University of Science and Technology of China, Shenyang 110016, China.

[2] Shenyang National Laboratory for Materials Science, Institute of Metal Research, Chinese Academy of Sciences, Shenyang 110016, China.

[3.]Shenzhen Key Lab of Energy Materials for Carbon Neutrality, Institute of Technology for Carbon Neutrality, Shenzhen Institute of Advanced Technology, Chinese Academy of Sciences, Shenzhen 518055, China.

[4.]Faculty of Materials Science and Energy Engineering, Shenzhen University of Advanced Technology, Shenzhen 518000, China.

*Corresponding author.

E-mail address: hcyang@imr.ac.cn; fli@imr.ac.cn; cheng@imr.ac.cn.

Table S1. The donor number, permittivity and $LiNO_3$ solubility of different solvents [1-8].

Solvents	DN (kcal mol^{-1})	Permittivity	$LiNO_3$ Solubility (mol L^{-1})
Ethylene carbonate (EC)	16.4	89.8	~0.7
Propylene carbonate (PC)	15.0	64.9	~0.2
Fluoroethylene carbonate (FEC)	9.1	78.4	<0.1
Dimethyl carbonate (DMC)	17.2	3.11	<0.1
Diethyl carbonate (DEC)	16	2.81	<0.1
Ethyl methyl carbonate (EMC)	/	2.96	<0.1
1,2-Dimethoxyethane (DME)	20	7.2	~0.4
Tetrahydrofuran (THF)	20	7.4	/
2-Methyltetrahydrofuran (2-Me-THF)	12	6.2	/
Acetonitrile (AN, ACN)	14	36.64	~0.3
1,3-Dioxolane (DOL)	21.2	7.1	/
Triethylamine (TEA)	61	2.42	<1.5
γ-Butyrolactone (γ-BL, GBL)	18	39	>2
Sulfolane (SL)	14.8	44	2.2
Trimethyl phosphate (TMP)	23	21.26	>4
Dimethyl sulfoxide (DMSO)	29.8	46.68	>4
Dimethylformamide (DMF)	26.6	36.7	>4
Tetramethylurea (TMU)	31.2	24.46	>4

The thermodynamic parameters of different electrolytes, such as binding energy, enthalpy of solvation, solvation entropy and solvation free energy, can be obtained more directly by theoretical calculation. For example, in the carbonate electrolyte of lithium batteries, the formulas as follows [9]:

$$\Delta E_b = E_{complex} - [nE_{(carbonate)} + E_{Li^+}] \text{ (S1)}$$

in which n, $E_{(carbonate)}$, E_{Li^+}, and $E_{complex}$ are the number of carbonate solvents and the energies of the carbonate solvent, Li^+, and complex, respectively. The enthalpy and free energy of solvation [$\Delta H_{(sol)}$ and $\Delta G_{(sol)}$, respectively] of the Li^+ by the carbonate solvents at 298.15 K were calculated by Formula S2:

$$\Delta X_{(sol)} = X_{complex} - [nX_{(carbonate)} + X_{Li^+}] \text{ (S2)}$$

in which X=H and G. The entropy (S) of solvation was also calculated at 298.15 K from Formula S3:

$$\Delta S_{(sol)} = \frac{[\Delta H_{(sol)} - \Delta G_{(sol)}]}{298.15} \text{ (S3)}$$

Based on quantum calculations of Fard et al [9], the thermodynamic variables of Li^+ with different carbonates are summarized as shown in Table S2.

Table S2. Binding energy (ΔE_b), enthalpy of solvation [$\Delta H_{(sol)}$], entropy of solvation [$\Delta S_{(sol)}$] and free energy of solvation [$\Delta G_{(sol)}$] in different carbonate electrolytes [9].

**Structure**	**ΔE_b [kcal mol^{-1}]**	**$\Delta H_{(sol)}$ [kcal mol^{-1}]**	**$\Delta G_{(sol)}$ [kcal mol^{-1}]**	**$\Delta S_{(sol)}$ [cal mol^{-1} K^{-1}]**
Li^+(EC)	-153.69	-164.39	-99.13	-217.88
Li^+(VC)	-122.79	-129.46	-79.55	-167.39
Li^+(PC)	-107.84	-109.34	-84.66	-82.78
Li^+(DMC)	-116.78	-124.32	-65.75	-196.45
Li^+(EMC)	-113.15	-117.99	-75.68	-141.91
Li^+(DEC)	-116.66	-123.05	-76.77	-155.20
Li^+(EC/PC)	-140.36	-148.28	-95.42	-177.29
Li^+(EC/DMC)	-143.96	-154.76	-98.20	-189.70
Li^+(EC/EMC)	-134.55	-143.95	-85.61	-195.66
Li^+(EC/DEC)	-135.99	-143.73	-89.22	-182.81

The lattice energies of some common metal (M=Li, Na, K) salts are shown in Table S3.

Table S3. Lattice energy (*U*, KJ mol^{-1}) of different metal salts [10].

**X**	**M**		
	Li	Na	K
Cl	821	748	690
Br	796	730	673
NO_3	823	741	685
SCN	780	700	648
CH_3CO_2	848	752	683
CF_3SO_3	735	654	598
CF_3CO_2	777	693	630
ClO_4	735	645	596
BF_4	737	667	614

Table S4. Electrolytes corresponding to different design strategies.

Design strategies	Type of electrolyte	Electrolytes	Ref.
**enthalpy**	Weak solvated electrolytes	1 M LiFSI-DMM	[11]
		LiFSI-CPME	[12]
		2.1 M LiFSI-F2EMP	[13]
	Anion coordination electrolytes	LiFSI-LiTFA-DEE	[14]
		1 M LIFPA-EC/DMC	[15]
		LiETFSI-$LiPF_6$-EC-DEC-FEC	[16]
**entropy**	High concentration electrolytes	4 M LiFSI-DME	[17]
		17 M LiFSI-DMC	[18]
		10 M LiFSI in EC-DMC	[19]
	Localized high concentration electrolytes	2 M LiFSI-EGBE-TTE (1:1 by volume)	[20]
		1.4 M LiFSI-DME-HFC (1:3 by mol)	[21]
		1.2 M LiFSI-DMC-BTFE (1:2 by mol)	[22]
	High entropy electrolytes	LiFSI-LiTFSI-LiDFOB-$LiNO_3$-$LiPF_6$-EC-DMC with 5% FEC	[2]
		LiFSI-LiTFSI-LiDFOB-$LiNO_3$-DME	[23]
		1 M LiFSI DME-DEE-DEGDME-TTE-BTFE	[24]

REFERENCES

1. Zhao Q, Utomo N W, Kocen A L *et al.* Upgrading carbonate electrolytes for ultra-stable practical lithium metal batteries. *Angew. Chem. Int. Ed.* 2022; **61**: e202116214. 10.1002/anie.202116214.

2. Wang Q, Zhao C, Yao Z *et al.* Entropy-driven liquid electrolytes for lithium batteries. *Adv. Mater.* 2023; **35**: 2210677. 10.1002/adma.202210677.

3. Piao N, Liu S, Zhang B *et al.* Lithium metal batteries enabled by synergetic additives in commercial carbonate electrolytes. *ACS Energy Lett.* 2021; **6**: 1839-1848. 10.1021/acsenergylett.1c00365.

4. Piao Z, Xiao P, Luo R *et al.* Constructing a stable interface layer by tailoring solvation chemistry in carbonate electrolytes for high-performance lithium-metal batteries. *Adv. Mater.* 2022; **34**: 2108400. 10.1002/adma.202108400.
5. Liu S, Ji X, Piao N *et al.* An inorganic-rich solid electrolyte interphase for advanced lithium-metal batteries in carbonate electrolytes. *Angew. Chem. Int. Ed.* 2021; **60**: 3661-3671. 10.1002/anie.202012005.
6. Zhou P, Hou W, Xia Y *et al.* Tuning and balancing the donor number of lithium salts and solvents for high-performance Li metal anode. *ACS Nano* 2023; **17**: 17169-17179. 10.1021/acsnano.3c05016.
7. Jie Y, Liu X, Lei Z *et al.* Enabling high-voltage lithium metal batteries by manipulating solvation structure in ester electrolyte. *Angew. Chem. Int. Ed.* 2020; **59**: 3505-3510. 10.1002/anie.201914250.
8. Zhou P, Xiang Y Liu K. Understanding and applying the donor number of electrolytes in lithium metal batteries. *Energy Environ. Sci.* 2024; **17**: 8057-8077. 10.1039/D4EE02989E.
9. Shakourian-Fard M, Kamath G, Sankaranarayanan S K R S. Evaluating the free energies of solvation and electronic structures of lithium-ion battery electrolytes. *ChemPhysChem* 2016; **17**: 2916-2930. 10.1002/cphc.201600338.
10. Kim C K, Won J, Kim H S *et al.* Density functional theory studies on the dissociation energies of metallic salts: relationship between lattice and dissociation energies. *J. Comput. Chem.* 2001; **22**: 827-834. 10.1002/jcc.1048.
11. Ma T, Ni Y, Wang Q *et al.* Optimize lithium deposition at low temperature by weakly solvating power solvent. *Angew. Chem. Int. Ed.* 2022; **61**: e202207927. 10.1002/anie.202207927.
12. Zhang H, Zeng Z, Ma F *et al.* Cyclopentylmethyl ether, a non-fluorinated, weakly solvating and wide temperature solvent for high-performance lithium metal battery. *Angew. Chem. Int. Ed.* 2023; **62**: e202300771. 10.1002/anie.202300771.
13. Wu L-Q, Li Z, Fan Z-Y *et al.* Unveiling the role of fluorination in hexacyclic coordinated ether electrolytes for high-voltage lithium metal batteries. *J. Am. Chem. Soc.* 2024; **146**: 5964-5976. 10.1021/jacs.3c11798.
14. Li J, Zhang J, Yu H *et al.* Designing high donor number anion additive for stable lithium metal batteries. *Small* 2024; **20**: 2408164. 10.1002/smll.202408164.
15. Li L, Xu G, Zhang S *et al.* Highly fluorinated Al-centered lithium salt boosting the interfacial compatibility of Li-metal batteries. *ACS Energy Lett.* 2022; **7**: 591-598. 10.1021/acsenergylett.1c02489.
16. Zhou P, Zhou H, Xia Y *et al.* Rational lithium salt molecule tuning for fast charging/discharging lithium metal battery. *Angew. Chem. Int. Ed.* 2024; **63**: e202316717. 10.1002/anie.202316717.
17. Qian J, Henderson W A, Xu W *et al.* High rate and stable cycling of lithium metal anode. *Nat. Commun.* 2015; **6**: 6362. 10.1038/ncomms7362.
18. Lu Z, Yang H, Sun J *et al.* Conformational isomerism breaks the electrolyte solubility limit and stabilizes 4.9 V Ni-rich layered cathodes. *Nat. Commun.* 2024; **15**: 9108. 10.1038/s41467-024-53570-1.

19. Fan X, Chen L, Ji X *et al.* Highly fluorinated interphases enable high-voltage Li-metal batteries. *Chem* 2018; **4**: 174-185. 10.1016/j.chempr.2017.10.017.
20. Jie Y, Wang S, Weng S *et al.* Towards long-life 500 Wh kg^{-1} lithium metal pouch cells via compact ion-pair aggregate electrolytes. *Nat. Energy* 2024; **9**: 987-998. 10.1038/s41560-024-01565-z.
21. Wu Z, Li R, Zhang S *et al.* Deciphering and modulating energetics of solvation structure enables aggressive high-voltage chemistry of Li metal batteries. *Chem* 2023; **9**: 650-664. 10.1016/j.chempr.2022.10.027.
22. Chen S, Zheng J, Mei D *et al.* High-voltage lithium-metal batteries enabled by localized high-concentration electrolytes. *Adv. Mater.* 2018; **30**: 1706102. 10.1002/adma.201706102.
23. Wang Q, Zhao C, Wang J *et al.* High entropy liquid electrolytes for lithium batteries. *Nat. Commun.* 2023; **14**: 440. 10.1038/s41467-023-36075-1.
24. Kim S C, Wang J, Xu R *et al.* High-entropy electrolytes for practical lithium metal batteries. *Nat. Energy* 2023; **8**: 814–826. 10.1038/s41560-023-01280-1.
